# Capsular contractures following implant-based breast reconstruction in women undergoing risk-reducing mastectomy: national register-based study

**DOI:** 10.1093/bjsopen/zraf080

**Published:** 2025-07-29

**Authors:** Signe Hägglund, Johan Svensson, Emma Hansson, Martin Halle, Rebecca Wiberg

**Affiliations:** Department of Diagnostics and Intervention, Plastic Surgery and Surgery, Umeå University, Umeå, Sweden; Department of Statistics, Umeå School of Business, Economics and Statistics, Umeå University, Umeå, Sweden; Department of Plastic Surgery, Institute of Clinical Sciences, Sahlgrenska Academy, University of Gothenburg, Gothenburg, Sweden; Department of Plastic and Reconstructive Surgery, Sahlgrenska University Hospital, Region Västra Götaland, Gothenburg, Sweden; Department of Molecular Medicine and Surgery, Karolinska Institutet, Stockholm, Sweden; Department of Reconstructive Plastic Surgery, Karolinska University Hospital, Stockholm, Sweden; Department of Diagnostics and Intervention, Plastic Surgery and Surgery, Umeå University, Umeå, Sweden

## Abstract

**Background:**

The majority of women undergoing risk-reducing mastectomy have implant-based breast reconstruction, with capsular contracture being one of the most common complications. The primary aim of this study was to establish the national incidence rate of severe capsular contracture requiring surgery following risk-reducing mastectomy with implant-based breast reconstruction. The secondary aim was to establish the incidence rate of other complications and associated risk factors.

**Methods:**

Women undergoing implant-based breast reconstruction following risk-reducing mastectomy were identified from the Swedish Breast Implant Register. Data were extracted from the Swedish Breast Implant Register and the National Patient Register on women undergoing implant-based breast reconstruction from 2014 to 2021. The primary outcome was severe capsular contracture corresponding to Baker grade III–IV requiring surgery, and the secondary outcomes were other complications observed perioperatively.

**Results:**

In total, 656 women with 1095 implant-based breast reconstructions were included in the analysis. Median follow-up was 3.5 (interquartile range 1.5–5.4) years. Capsular contracture was observed in 39 of 1095 breasts (3.6%), and the cumulative incidence increased from 1.9% at 1 year to 4.7% after 5 years. Stratified by implant type, the estimated risk of capsular contracture increased for patients with a permanent tissue expander compared with a permanent fixed-volume implant (adjusted hazard ratio 19.33, 95% confidence interval 3.92 to 95.43; *P* < 0.001).

**Conclusion:**

This study has highlighted that the risk of developing severe capsular contracture requiring surgery seems to differ between implant types, emphasizing the need for further investigation regarding permanent tissue expanders. Moreover, the continuous increase in capsular contracture incidence rates over 5 years underscores the importance of long-term follow-up.

## Teaser

The primary aim of this study was to establish the national incidence rate of severe capsular contracture requiring surgery following risk-reducing mastectomy with implant-based breast reconstruction. The secondary aim was to establish the incidence rate of other complications and associated risk factors. In total, 656 women with 1095 breast reconstructions were included in the analysis with a median follow-up of 3.5 (interquartile range 1.5–5.4) years. The cumulative incidence of capsular contracture increased from 1.9% at 1 year to 4.7% after 5 years. Stratified by implant type, the estimated risk of capsular contracture increased for patients with a permanent tissue expander compared with a permanent fixed-volume implant.

## Introduction

With an improved diagnosis of genetic predispositions for breast cancer during the past decades^1^, risk-reducing mastectomies (RRMs) in previously healthy women have increased^2–4^; 32–48% of all cancer-free *BRCA1/2* carriers have been reported to undergo bilateral RRM^5,6^. In addition, contralateral RRM after breast cancer in women without pathological gene variants has increased^6,7^, although there is notable variation across European countries and the USA. In the USA, around one in four women undergo contralateral RRM in addition to therapeutic mastectomy^6,7^, whereas in Sweden, only women with high-penetrance breast cancer genes are recommended to undergo contralateral RRM^8^.

Since the introduction of silicone breast implants in 1962, implant-based breast reconstruction (IBBR) has been the most common technique for breast reconstruction following RRM; only a minority of all women undergo autologous tissue reconstruction or opt for no reconstruction at all^9–12^. IBBR may be achieved in one stage comprising direct-to-implant reconstruction with a permanent fixed-volume implant (permanent implant) or a permanent tissue expander (TE), or in two stages using a temporary TE followed by a permanent implant at a later stage. A permanent TE containing cohesive silicone gel in the outer chamber and normal saline in the inner chamber was introduced to eliminate the need to replace a temporary TE with a permanent implant^13^.

However, implants are associated with several complications, including haematoma, infection, implant rupture, rotation, malposition, capsular contractures, autoimmune/inflammatory syndrome, and anaplastic large-cell lymphoma^14–16^. Capsular contracture is one of the most common long-term complications and reasons for reintervention^17–21^. The pathogenesis of capsular contractures is thought to be multifactorial. Several risk factors have been suggested in the literature, including prepectoral placement (compared with subpectoral)^17,18,22,23^, use of a silicone-filled implant (compared with saline-filled)^18,24^, a smooth surface (compared with textured)^18,22–25^, having a breast reconstruction after mastectomy (compared with cosmetic augmentation)^17,20,26^, having a one-stage breast reconstruction (compared with two-stage)^19^, and radiotherapy (compared with no radiotherapy)^27,28^. The reported incidence rate of capsular contracture following IBBR varies between 2.3 and 39%^17–20,24,25,29–32^, presumably depending on heterogeneity in follow-up time, indications, surgical techniques, use of radiotherapy, and a subjective classification system, creating a need for long-term follow-up studies on homogeneous groups of patients.

In Sweden, most implants are registered in a national register, the Swedish Breast Implant Register (BRIMP)^33^, which has been fully implemented since 2014. In BRIMP, capsular contracture is defined as a perioperative finding of a dense capsule of connective tissue around the implant requiring surgery^34^. Although the register has been in place for 10 years, the data have never been used to scientifically evaluate the capsular contracture rate after breast reconstruction. Using data from a register could be a way of avoiding the methodological pitfalls of previous studies described above.

The primary aim of this study was to establish the national incidence rate of severe capsular contracture requiring surgery following RRM with IBBR. The secondary aim was to establish the incidence rate of other complications and associated risk factors.

## Methods

### Patient cohort

Women undergoing IBBR after mastectomy, with the breast reconstruction performed between 1 January 2014 and 31 December 2021, were identified from BRIMP^33^. Inclusion criteria were women undergoing IBBR following RRM with permanent implants and permanent TEs. Exclusion criteria were therapeutic mastectomy, previous ipsilateral breast tumour, radiotherapy before breast reconstruction, and IBBR with temporary TEs. In total, 2125 women and 3006 breasts were identified from BRIMP. Because BRIMP does not provide information on when the mastectomy was performed, the Patient Register was used to determine whether the IBBR was a one- or two-stage procedure. Variables from the register considered as primary and secondary outcomes were severe capsular contracture, rotation, migration, rupture, infection, seroma, and haematoma, all requiring surgical intervention, as well as permanent implant extraction independently of the aetiology. Definitions of outcomes according to BRIMP are described below. The follow-up period was defined as the interval between IBBR and capsular contracture, death, permanent implant extraction, or end of follow-up (31 December 2021), whichever event came first.

### The BRIMP register

BRIMP^33^ is a national quality register with national data on women undergoing IBBR or breast augmentation for other indications registered prospectively since its establishment in 2014. Only permanent implants and TEs, not temporary TEs, are intended to be registered In BRIMP^34^. The variables are reported by the surgeon at the time of primary breast reconstruction and reoperation, if any. The variables comprise patient data, implant-related data, surgical techniques, reasons for revision surgery, perioperative status at reoperation, and type of surgical intervention. BRIMP has an affiliation with all Swedish university clinics in plastic surgery, in addition to over 85% of all plastic surgeons practising privately in Sweden^34^, with 65% of all sold implants registered in BRIMP^35^. The register is, however, yet to be validated.

### National Patient Register

The National Patient Register in Sweden^36^ contains information on all completed inpatient stays since 1964 (nationwide since 1987), day surgery since 1997, and other specialized outpatient care since 2001. The coverage of the register is generally regarded to be high^37^ and, despite lacking comparative data, the positive predictive value has been reviewed as high^38^. Data are extracted from medical records reported by healthcare providers and comprise diagnoses based on International Classification of Disease codes, and surgical procedures based on the National Classification of Health Care Interventions (KVÅ; 1997–present)^39^ and the National Classification of Operations (1963–1996). Administrative data relating to inpatients covers dates for admission and discharge, and duration of hospital stay^37^.

### Definitions

The type of permanent implant or TE used in IBBR was defined according to the filling material reported in BRIMP, as either a permanent implant containing silicone or a permanent TE containing both silicone and saline. It is intended that BRIMP registers only permanent implants and TEs, not temporary TEs. However, permanent TEs containing saline only and incorrectly reported temporary TEs containing saline could not be distinguished; therefore, all implants and TEs containing saline only were excluded. Outcome variables were identified from the reoperation codes reported in BRIMP; thus, all reported outcomes required surgical intervention. Capsular contracture was defined as the perioperative finding of a dense capsule of connective tissue around the implant requiring surgery (T85.4—mechanical complication of breast prosthesis and implant), corresponding to Baker III or IV. Rupture, rotation, migration, seroma, and haematoma were all defined as observed conditions in perioperative status reported at reoperation. Postsurgical infection (T81.4) was defined as such if registered as a reason for reoperation. Permanent implant extraction was defined as implant removal without the insertion of a new implant during the same surgical procedure^40^. The implant placement was defined as either subpectoral or prepectoral, the latter including subfascial placement. Subpectoral placement includes both full and partial muscle coverage, although registered in BRIMP as two separate implant placements (subpectoral or dual plane, but here merged as a single variable). Further descriptions of included variables are available in *Table S1*.

In BRIMP, RRM before IBBR was identified through the indication ‘reconstruction following risk-reducing mastectomy’ in combination with ‘no previous ipsilateral breast tumour’ and ‘no previous radiotherapy’ at the time of IBBR. As the cohort was already established through BRIMP, RRM in the Patient Register considered any inpatient or outpatient episode with a registered nipple-sparing mastectomy (KVÅ: HAC10), skin-sparing mastectomy (HAC15), mastectomy (HAC20), or other mastectomy (HAC99). The date of RRM was subsequently merged with the BRIMP data set by matching the pseudonymized personal security numbers through the National Board of Health and Welfare. The timing of IBBR was defined as follows: IBBR registered within 30 days from the date of mastectomy was defined as one-stage breast reconstruction, whereas reconstruction performed more than 30 days after mastectomy was defined as two-stage reconstruction. It was not possible to distinguish between two-stage IBBR and delayed IBBR; therefore, all IBBRs performed more than 30 days after mastectomy were considered two-stage reconstructions, with the possibility that some delayed one-stage breast reconstructions were included erroneously in the group. If more than one RRM code was present in the same individual with separate dates, the most recent date before the IBBR was considered the RRM date, given that the first mastectomy referred to a contralateral therapeutic procedure following breast cancer that was excluded from the study sample through BRIMP. Women with RRM performed after the IBBR date were excluded from the study cohort, whereas those without a recorded date for RRM in the Patient Register were treated as missing data, as some patients might have undergone surgery at a clinic outside the hospital.

### Ethical considerations

The study was carried out according to the principles of the Declaration of Helsinki and the ethical guidelines of the Swedish Research Council. Ethical approval was obtained from the National Ethical Review Board in Sweden (2021-00953). Informed consent was waived as the study was based on register-based data.

### Statistical analysis

All statistical analyses were conducted per breast/IBBR rather than per patient. Descriptive data on patient characteristics, implant characteristics, and frequencies of complications and permanent implant extractions are presented as numbers with percentages, mean(s.d.) values, and median (interquartile range, i.q.r.) values. The cumulative incidence of severe capsular contracture requiring surgery was analysed with Kaplan–Meier estimates and 95% confidence intervals (c.i.). Capsular contracture was defined as the event, whereas death, permanent implant extraction, and end of follow-up were defined as censures. A Cox frailty model was used to conduct hazard inference in the dependent data structures arising from some breasts belonging to the same women, presented as hazard ratios (HRs) with 95% c.i. In this model, two breasts in the same woman were considered to share frailty. The proportional hazards assumptions were assessed visually. Initially, univariable analyses were undertaken, and variables significantly associated with capsular contracture and the timing of reconstruction were subsequently included in multivariable analyses. Owing to the small number of capsular contractures and the frailty assumption, only limited adjustments could be made. Cox regression analyses regarding reoperations were conducted with first reoperation as the endpoint. Multiple imputation in 20 data sets estimated with chained equations was used to handle missing data. As an imputation model, variables in the Cox models were used together with the event status variable and Nelson–Aalen estimates of the cumulative hazard^41^. Missing data on insertion of net or acellular dermal matrix were not imputed because of the large number of missing values. A polyurethane-covered surface was excluded from Cox analyses because it was registered only once. The significance level was set at 0.050. Statistical analyses were carried out using SPSS^®^ for Macintosh^®^ version 29.0 (IBM, Armonk, NY, USA) and Stata^®^ release 17.0 (StataCorp, College Station, TX, USA).

## Results

### Study cohort

**Fig. 1:**
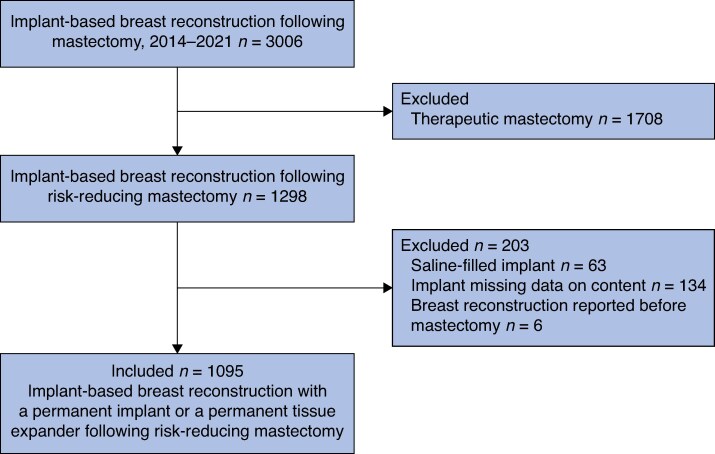
Study flow chart

In total, 2125 women with 3006 breasts underwent IBBR following mastectomy, of whom 781 women with 1298 breasts underwent IBBR following RRM during the study period. Sixty-three breasts were excluded because the implant content was reported as saline only, 134 were excluded owing to missing data on implant content, and 6 breasts were excluded because IBBR was performed before the RRM. After exclusions, a total of 656 women with 1095 breasts were included in the analysis (*Fig. 1*), with a median follow-up of 3.5 (i.q.r. 1.5–5.4) years per breast. The median age at breast reconstruction was 40.6 (i.q.r. 34.1–48.5) years; half of the IBBRs were performed in women aged ≤ 40 years (562 of 1095, 51.3%). The median body mass index (BMI) was 23.2 (i.q.r. 21.4–25.7) kg/m^2^, and BMI was < 25 kg/m^2^ in 686 of 1095 IBBRs (missing data: 104 breasts, 9.5%). Among 1095 IBBRs, the breast reconstruction involved a permanent implant in 770 breasts (70.3%) and a permanent TE in 325 (29.7%). Most IBBRs were performed as one-stage procedures (916 breasts, 83.7%) and there were 134 two-stage IBBRs (12.2%) (missing data: 45 breasts, 4.1%) (*Table 1*). Permanent implants were more commonly smaller than 350 ml (565 of 770, 73.4%) (missing data: 12 breasts, 1.1%), whereas permanent TEs were more frequently larger than 350 ml (205 of 325, 63.1%) (missing data: 57 breasts, 17.5%). Surgical characteristics are presented in *Table 1*.

**Table 1:** Surgical characteristics of implant-based breast reconstruction after risk-reducing mastectomy

	No. of breasts* (*n* = 1095)
**Type of implant**	
Permanent implant	770 (70.3%)
Permanent tissue expander	325 (29.7%)
**Timing of breast reconstruction**	
One-stage IBBR	916 (83.7%)
Two-stage IBBR	134 (12.2%)
**Placement**	
Subpectoral†	1012 (92.4)
Prepectoral‡	63 (5.8)
**Volume of permanent implant (ml)**	
Mean(s.d.)	313(89)
Median (i.q.r.)	300 (255–360)
**Volume of permanent tissue expander (ml)**	
Mean(s.d.)	398(100)
Median (i.q.r.)	400 (365–460)
**Manufacturer**	
Mentor	1055 (96.3%)
Allergan	24 (2.2%)
Other	14 (1.3%)
**Surface**	
Microtextured or macrotextured	940 (85.8%)
Smooth or nanotextured	141 (12.9%)
Polyurethane covered	1 (0.1%)
**Shape**	
Anatomical	872 (79.6%)
Round	207 (18.9%)
**Preoperative or perioperative prophylactic antibiotic treatment**	
No	50 (4.6%)
Yes	1009 (92.1%)
**Postoperative prophylactic antibiotic treatment**	
No	570 (52.1%)
Yes	474 (43.3%)
**Insertion of drains**	
No	111 (10.1%)
Yes	823 (75.2%)
**Insertion of net or ADM**	
No	398 (36.3%)
Yes	133 (12.1%)

*Values are number of breasts (%) unless otherwise indicated. Where the total percentage for a variable is less than 100%, the difference represents the proportion of missing values. †Includes total or partial muscle coverage (dual plane). ‡Includes prepectoral and subfascial placement. IBBR, implant-based breast reconstruction; s.d., standard deviation; i.q.r., interquartile range; ADM, acellular dermal matrix. Mentor (Mentor Worldwide LLC, Irvine, CA, USA). Allergan (Allergan plc, Dublin, Ireland) .

### Capsular contractures

**Fig. 2:**
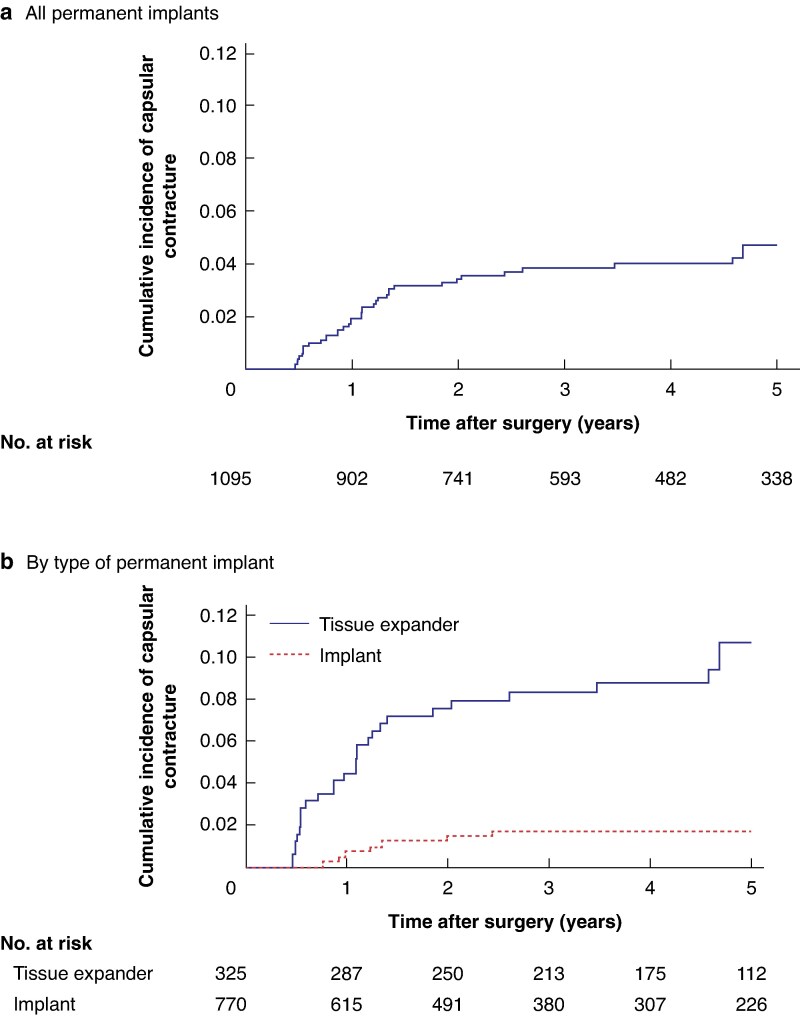
Cumulative incidence of severe capsular contracture requiring surgery over time **a** Among all implants and **b** stratified by type of permanent implant. The cumulative incidence of severe capsular contracture requiring surgery was analysed using Kaplan–Meier estimates over 5 years.

Among 1095 IBBRs, severe capsular contracture requiring surgery was observed in 39 breasts (3.6%) in 30 women; 18 were bilateral capsular contractures, 7 were unilateral capsular contractures in women with both breasts having undergone IBBR following RRM, and 14 were unilateral capsular contractures in women with only 1 breast subjected to IBBR following RRM. The cumulative incidence of capsular contracture was 1.9 (95% c.i. 1.2 to 2.9)% at 1 year, 3.4 (95% c.i. 2.4 to 4.8)% at 2 years, 3.9 (95% c.i. 2.8 to 5.4)% at 3 years, 4.0 (95% c.i. 2.9 to 5.6)% at 4 years, and 4.7 (95% c.i. 3.4 to 6.5)% after 5 years. The majority of capsular contractures (32 of 39, 82%) occurred within 2 years after the breast reconstruction (*Fig. 2a* and  *Table S2*). The cumulative incidence of capsular contracture increased continuously up to 5 years after IBBR, and after that remained constant between years 5 and 8 (*Fig. 2a* and  *Table S2*).

### Factors associated with capsular contractures

In univariable analyses of risk factors for capsular contracture, the use of a permanent TE (HR 32.98, 95% c.i. 6.75 to 161.08; *P* < 0.001) and postoperative prophylactic antibiotic treatment (HR 0.18, 95% c.i. 0.04 to 0.89; *P* = 0.035) were the only factors with a significant association with the development of capsular contractures (*Table 2*). The timing of breast reconstruction (one- *versus* two-stage), age at IBBR, implant surface, implant shape, implant placement, implant volume, BMI, insertion of drains, and insertion of net or acellular dermal matrix were not associated risk factors (*Table 2*). However, in a multivariable model with predictors (permanent TE, postoperative prophylactic antibiotic treatment, and timing of breast reconstruction), the statistically significant association between postoperative antibiotic treatment and capsular contracture was lost, the lack of association with timing of breast reconstruction remained, and only the use of a permanent TE showed a significant association with the risk of capsular contracture (HR 19.33, 95% c.i. 3.92 to 95.43; *P* < 0.001) (*Table 2*). Implant surface and placement were added to an additional multivariable analysis to rule out the impact of risk factors previously identified in the literature. The use of a permanent TE remained significantly associated with the development of capsular contracture (HR 23.84, 95% c.i. 4.72 to 120.52; *P* < 0.001) even when adjusting for implant surface, implant placement, and previously mentioned predictors. Volume of the implant was not included because the model was overparametrized when this variable was included. When stratified by implant type, the cumulative incidence of capsular contracture over 5 years was 1.7 (95% c.i. 0.9 to 3.1)% for permanent implants and 10.8 (95% c.i. 7.5 to 15.3)% for permanent TEs (*Fig. 2b*).

**Table 2:** Factors associated with severe capsular contractures requiring surgery identified by frailty Cox proportional hazards regression survival analysis

	Unadjusted analysis		Adjusted analysis	
	Hazard ratio	*P*	Hazard ratio	*P*
**Timing of breast reconstruction**				
One-stage IBBR	1.00 (reference)		1.00 (reference)	
Two-stage IBBR	0.13 (0.01, 3.11)	0.209	0.35 (0.02, 4.86)	0.432
**Type of implant**				
Permanent implant	1.00 (reference)		1.00 (reference)	
Permanent tissue expander	32.98 (6.75, 161.08)	< 0.001	19.33 (3.92, 95.43)	< 0.001
**Age (years)**				
≤ 40	1.00 (reference)			
> 40	1.77 (0.35, 9.06)	0.494		
**Surface**				
Microtextured or macrotextured	1.00 (reference)			
Smooth or nanotextured	0.44 (0.03, 5.62)	0.530		
**Shape**				
Anatomical	1.00 (reference)			
Round	0.40 (0.05, 3.09)	0.376		
**Placement**				
Subpectoral*	1.00 (reference)			
Prepectoral†	1.45 (0.07, 29.26)	0.808		
**Implant volume (ml)**				
≤ 350	1.00 (reference)			
> 350	3.97 (0.58, 27.28)	0.161		
**BMI (kg/m^2^)**				
< 25	1.00 (reference)			
≥ 25	0.90 (0.16, 4.99)	0.908		
**Prophylactic postoperative antibiotic treatment**				
No	1.00 (reference)		1.00 (reference)	
Yes	0.18 (0.04, 0.89)	0.035	0.53 (0.11, 2.55)	0.428
**Insertion of drains**				
No	1.00 (reference)			
Yes	2.09 (0.16, 26.60)	0.571		
**Insertion of net or ADM**‡				
No	1.00 (reference)			
Yes	0.56 (0.09, 3.66)	0.543		

Values in parentheses are 95% confidence intervals. The number of breasts in each group may have differed because of missing data on a specific factor. *Includes total or partial muscle coverage (dual plane). †Includes prepectoral and subfascial placement. ‡Missing values in insertion of net or acellular dermal matrix were not imputed because of high proportion of missing values. IBBR, implant-based breast reconstruction; BMI, body mass index; ADM, acellular dermal matrix. *P* <0.050 was considered statistically significant.

### Surgical intervention for capsular contractures

Regarding surgical treatment of the 39 capsular contractures, capsulotomy was performed in 31 breasts, partial capsulectomy in 5, total capsulectomy in 1, and *en bloc* resection in 1. Surgical interventions at reoperation were neither compulsory to register nor restricted to a single intervention. Regarding management of the implant, it was exchanged with a new implant in 35 breasts, permanently removed in 2, and removed with reinsertion of the same implant after treatment in 2 breasts. Fat transplantation at reoperation was registered in three breasts.

### Factors associated with reoperation

Of 1095 IBBRs, 159 breasts underwent a single reoperation (14.5%), 13 underwent 2 reoperations (1.2%), and 6 underwent 3 reoperations (0.5%), resulting in 178 breasts (16.3%) in 125 women undergoing at least 1 additional surgery following IBBR. Both permanent TE use (HR 4.06, 95% c.i. 1.75 to 9.44; *P* = 0.001) and one-stage IBBR (HR 5.59, 95% c.i. 1.22 to 25.55; *P* = 0.026) increased the risk of having at least one reoperation in univariable analysis. However, in multivariable analysis accounting for both the timing of reconstruction and type of implant, permanent TE increased the risk of having at least one reoperation more than three-fold (HR 3.62, 95% c.i. 1.52 to 8.61; *P* = 0.004), whereas one-stage IBBR was no longer a significant risk factor (HR 3.41, 95% c.i. 0.87 to 13.31; *P* = 0.077).

### Rates of other complications

Of a total of 203 reoperations, the perioperative status revealed a capsular contracture in 39 breasts (19.2%), implant rotation in 23 (11.3%), migration in 20 (9.9%), seroma in 13 (6.4%), implant rupture in 9 (4.4%), and haematoma in 1 (0.5%). A diagnosed infection was the reason for surgical reoperation in 19 breasts (9.4%) undergoing reoperation. Registration of perioperative observations and reasons for surgical reoperation was neither compulsory nor restricted to a single observation or reason, and other perioperative observations and reasons were also optional at registration but were not further investigated in this report, explaining the total percentage not equalling 100%. In total, 178 of 203 reoperations (87.7%) had a registered reason for, or perioperative status findings, at reoperation.

### Permanent implant extraction

Among 1095 breast reconstructions, 18 breasts (1.6%) underwent permanent implant extraction. Permanent implant extraction was carried out because of postoperative infection in 13 breasts, patient-experienced swelling in 7 breasts, the patient's desire for implant removal in 7 breasts, patient-experienced hardness of the breast in 4 breasts, patient-experienced pain in 4 breasts, patient's worry about the implant or its position in 2 breasts, and size or shape change desired by the patient in 1 breast. No woman underwent permanent implant extraction owing to breast implant illness or a newly diagnosed breast cancer during follow-up. As with other complications, registration of reasons for permanent implant extraction was neither compulsory nor restricted to a single reason.

## Discussion

This is the first national study to evaluate severe capsular contractures requiring surgery after RRM and IBBR using register-based data in Sweden. The main findings are that most capsular contractures develop within 2 years of the immediate reconstruction and the incidence continues to increase for at least 5 years after the primary operation, with the risk being significantly associated with type of implant.

A register-based study is inherently limited by the quality of the data in the register. First, given that IBBR after RRM is essentially being managed in public healthcare and that all Swedish university clinics in plastic surgery are affiliated with BRIMP, high coverage in the current field is expected. However, the BRIMP register is yet to be validated to increase its reliability and to determine the actual level of coverage. The second factor worth discussing is the definition of capsular contracture. There is no generally accepted and validated method for defining, diagnosing, or classifying capsular contractures^42,43^. The most widely used classification of capsular contracture, that of Baker^44^, has never been validated or reliability tested and entails considerable subjectiveness^45^. In the BRIMP register and this study, capsular contracture was defined as the perioperative observation of a dense, thickened capsule, corresponding to Baker III or IV. However, this definition is also subjective, as different patients and surgeons have varying thresholds for considering that an operation is indicated, and it does not necessitate the presence of a dense fibrous tissue capsule around the implant. Moreover, some surgeons might register capsular contracture as a perioperative finding in all breasts requiring some capsular modification, such as capsulotomy owing to malposition of the implant^29,46^, giving a falsely high capsular contracture rate. On the other hand, a number of factors might have contributed to a falsely low capsular contracture rate, such as patients yet to undergo corrective surgery for various reasons, namely long waiting time for surgery, which is a growing problem in Sweden, patients not seeking medical care, and patients undergoing primary breast reconstruction shortly before the end of follow-up in this study.

In the present study, after a median follow-up of 3.5 (i.q.r. 1.5–5.4) years, severe capsular contracture was observed in 39 of 1095 breasts (3.6%). Bilateral capsular contracture was observed in 18 of 39 breasts, a proportion similar to that reported in previous studies^47^. As speculated by others^47^, bilateral presentation suggests a partly patient-related aetiology. However, further statistical analysis was not possible owing to the limited number of capsular contractures. The cumulative incidence of capsular contracture increased from 1.9 (95% c.i. 1.2 to 2.9)% after 1 year to 4.7 (95% c.i. 3.4 to 6.5)% at 5 years after IBBR. The majority of capsular contractures occurred within 2 years after breast reconstruction, and the incidence continued to increase up to 5 years after the primary operation. Previous studies with long-term follow-up reported capsular contracture rates similar to those in the present study; an American study^48^ including 99 993 patients with 7-year follow-up reported a capsular contracture rate of 5.0% for silicone implants and 2.8% for saline implants, and an Australian study^19^ comprising 5152 breast reconstructions with 2-year follow-up reported a capsular contracture rate of 3.1% (161 of 5152). The third factor that needs to be mentioned is that the reasons for surgical reoperation were neither compulsory to register nor restricted to a single observation or reason. In total, 178 of all 203 reoperations (87.7%) had a registered reason or a perioperative finding, whereas 12.3% lacked an aetiology for reoperation, which might have affected the number of capsular contractures identified.

Regarding risk factors associated with capsular contractures, prophylactic postoperative antibiotic treatment (HR 0.18, 95% c.i. 0.04 to 0.89; *P* = 0.032) and permanent TE (HR 32.98, 95% c.i. 6.75 to 161.08; *P* < 0.001) were significantly associated with the risk of capsular contracture in univariable analysis. The rate of postoperative prophylactic antibiotic treatment was surprisingly high, given in almost half of IBBRs, despite previous literature stating that multiple-dose antibiotic prophylaxis is not superior to a single-dose regimen in preventing implant removal owing to surgical site infection^49^. However, in multivariable analysis, only permanent TE remained significantly associated with capsular contracture development. Permanent TEs are frequently used in IBBR^10,12,50^, both owing to the benefit of avoiding a second surgical procedure, and because current data indicate that radiotherapy should be carried out on the final implant rather than a temporary TE^27^. The literature regarding postoperative complications following use of permanent TEs is, however, scarce. Scuderi *et al.*^51^ observed capsular contractures in only 2.4% of 248 breasts reconstructed with Becker implants, a type of permanent TE, although the short follow-up of approximately 1 year made the assessment difficult. The increased risk of capsular contracture following IBBR with a permanent TE was hypothesized to be due to the timing of the reconstruction rather than the implant type itself, but data registered in BRIMP do not reveal whether the reconstruction was performed as an immediate or delayed procedure or in one or two stages. For that reason, additional information was collected from the Patient Register, but data matching was challenging regarding the staging of reconstructions, and some crude definitions were therefore needed to enable classification into one- or two-stage IBBR. Adjusting for one- *versus* two-stage reconstruction did not affect the outcome, and neither did adjustment for other well known risk factors from the literature such as implant placement (subpectoral *versus* prepectoral) and surface of the implant (textured *versus* smooth), with permanent TE remaining a significant risk factor for capsular contracture (HR 23.84, 95% c.i. 4.72 to 120.52; *P* < 0.001). However, the majority of breast implants had the same placement, manufacturer, surface and shape, which, together with a limited number of capsular contractures restricted the number of feasible variables in multivariable analysis, making it difficult to establish causation. Furthermore, the variable used for radiotherapy was based on radiotherapy before IBBR, resulting in a small risk of selection bias, considering that permanent TEs are more often used in the setting of adjuvant radiotherapy, although the inclusion of RRMs and exclusion of therapeutic mastectomies should have minimized that risk. Thus, the high frequency of capsular contractures associated with permanent TEs in this study must be interpreted with caution.

This study has highlighted that the risk of developing severe capsular contracture requiring surgery seems to be higher with use of permanent TEs than with permanent implants, emphasizing the need for further investigation in the field of permanent TEs. In addition, the continuous increase in the incidence of capsular contracture requiring surgery over 5 years underscores the importance of long-term follow-up.

## Supplementary Material

zraf080_Supplementary_Data

## Data Availability

The data that support the findings of this study are not openly available due to reasons of sensitivity but are available from the corresponding author upon reasonable request.
