# Supplementary material for: Capsular contractures following implant-based breast reconstruction in women undergoing risk-reducing mastectomy: national register-based study
Source: BJS Open. 2025 Jul 29;9(4):zraf080. doi: 10.1093/bjsopen/zraf080 (PMC12305423; doi:10.1093/bjsopen/zraf080)
Supplement: zraf080_Supplementary_Data [file zraf080_supplementary_data.docx]

**Capsular contractures following implant-based breast reconstruction in women undergoing risk-reducing mastectomy – a national register-based study**

Signe Hägglund MD^1*^, Johan Svensson PhD^2^**,** Emma Hansson MD, PhD^3,4^, Martin Halle MD, PhD^5,6^, Rebecca Wiberg MD, PhD^1^

^1^ Department of Diagnostics and Intervention, Plastic Surgery and Surgery, Umeå University, Umeå, Sweden.

^2^ Department of Statistics, Umeå School of Business, Economics and Statistics, Umeå University, Umeå, Sweden.

^3^ Department of Plastic Surgery, Institute of Clinical Sciences, Sahlgrenska Academy, University of Gothenburg, Gothenburg, Sweden.

^4^ Department of Plastic and Reconstructive Surgery, Sahlgrenska University Hospital, Region Västra Götaland, Gothenburg, Sweden.

^5^ Department of Molecular Medicine and Surgery, Karolinska Institutet, Stockholm, Sweden.

^6^ Department of Reconstructive Plastic Surgery, Karolinska University Hospital, Stockholm, Sweden.

^*^ **Corresponding author**:

Signe Hägglund

Department of Diagnostics and Intervention, Plastic Surgery and Surgery, Umeå University, Umeå, Sweden

Umeå University, SE-901 87 Umeå

[signe.hagglund@umu.se](mailto:signe.hagglund@umu.se)

<https://orcid.org/0009-0008-1691-0857>

**Supplementary Materials - Index**

|  |  |
| --- | --- |
| **Supplementary Figures and Tables** |  |
| Supplementary Table 1 | *page 2–4* |
| Supplementary Table 2 | *page 5* |
|  |  |

**Supplementary Figures and Tables**

**Supplementary Table 1. Variable description.** Description of the variables used in the present study, obtained from the Swedish Breast Implant Register.

| **Supplementary Table 1. Variable description** | | |
| --- | --- | --- |
| **Variable** | **Variable description** | **Outcome** |
| ***Breast reconstruction*** |  |  |
| Date of breast reconstruction | The date of the breast reconstruction in the format YYYY-MM-DD |  |
| Age at breast reconstruction | The woman’s age at breast reconstruction in years |  |
| BMI at breast reconstruction | Calculated from patient-reported length and weight at breast reconstruction |  |
| Prophylactic antibiotic treatment | Indicates whether the patient has received prophylactic (pre- or perioperative) antibiotic treatment before breast surgery | No |
|  |  | Yes |
| Intraoperative antibiotic treatment | Indicates whether the patient has received antibiotic treatment intraoperatively (implant irrigation or cavity irrigation) during the breast reconstruction | No |
|  |  | Yes |
| Postoperative antibiotic treatment | Indicates whether the patient are going to have prophylactic antibiotic treatment after breast reconstruction | No |
|  |  | Yes |
| Side | Indicates which side this breast reconstruction registration applies to | Right |
|  |  | Left |
| Operation indication | Indication for breast reconstruction. Reconstruction after risk-reducing mastectomy includes no previous ipsilateral breast cancer, no previous ipsilateral breast surgery due to tumour, and no previous history of radiation therapy to the breast or chest before the breast reconstruction. Reconstruction following therapeutic mastectomy includes positive answer to any of the above. | Reconstruction following cancer |
|  |  | Reconstruction following risk-reducing mastectomies |
| Implant type | Type of implant inserted for reconstruction during breast reconstruction based on the implant’s content, with silicone filling being considered a permanent implant, and combined silicone and saline filling being considered a permanent tissue expander | Permanent implant |
|  |  | Permanent tissue expander |
| Manufacturer of implant | Manufacturer of permanent implant or permanent tissue expander used in breast reconstruction | Allergan |
|  |  | Mentor |
|  |  | Others (Silimed, Motiva, Others) |
| Content of implant | Chemical filling material of the implant inserted during breast reconstruction | Saline and silicone |
|  |  | Silicone |
|  |  | Saline solution |
| Volume of implant | Volume of the permanent implant or permanent tissue expander inserted during breast reconstruction (cc, ml, gram) |  |
| Surface of implant | Type of surface of permanent implant or permanent tissue expander inserted during breast reconstruction | Smooth or nano-textured |
|  |  | Micro- or macro-textured |
|  |  | Polyurethane covered |
| Shape of implant | Shape of permanent implant or permanent tissue expander inserted during breast reconstruction | Round |
|  |  | Anatomic |
| Position of implant | Position of permanent implant or permanent tissue expander inserted at breast reconstruction | Subpectoral (Subpectoral, Dual plane) |
|  |  | Prepectoral (Prepectoral, Subglandular, Subfascial) |
| Insertion of drainage | Whether a drainage was inserted at the breast reconstruction | No |
|  |  | Yes |
| Insertion of net or acellular dermal matrix | Whether a net or an acellular dermal matrix was inserted at the breast reconstruction | No |
|  |  | Yes |
| ***Reoperation*** |  |  |
| Date of reoperation | The date of the reconstruction in the format YYYY-MM-DD |  |
| Age at reoperation | The woman’s age at reoperation in years |  |
| Side | Indicates which side this reoperation registration applies to | Right |
|  |  | Left |
| Pain | Patient-experienced pain in the breast is reason to the registered reoperation | No |
|  |  | Yes |
| Swelling of breast | Patient-experienced swelling in the breast is reason to the registered reoperation | No |
|  |  | Yes |
| Worry for implant or its position | Patient-experienced worry for the inserted implant or its position is reason to the registered reoperation | No |
|  |  | Yes |
| Desired size or shape change | Patient’s desire for size or shape change is reason to the registered reoperation | No |
|  |  | Yes |
| Hard breast | Patient-experienced hardness of the breast is reason to the registered reoperation | No |
|  |  | Yes |
| Desired implant removal | Patient’s desire of implant removal is reason for the registered reoperation | No |
|  |  | Yes |
| Infection (T81.4) | Postoperative infection, T81.4, is reason for the registered reoperation | No |
|  |  | Yes |
| Newly diagnosed breast cancer | Patient’s breast cancer diagnosis is reason for the registered reoperation | No |
|  |  | Yes |
| Symptom complex Breast Implant Illness | Symptom complex Breast Implant Illness is reason for the registered reoperation | No |
|  |  | Yes |
| Rupture/Deflation | Perioperative status at reoperation: Damage in the outer envelope of the implant (from gap in the envelope to a dissolution state of implant shape). Volume and/or shape change in the implant/expander prosthesis due to loss of saline. | No |
|  |  | Yes |
| Rotation | Perioperative status at reoperation: The implant has rotated in the prosthetic cavity | No |
|  |  | Yes |
| Incorrect position/migration | Perioperative status at reoperation: The implant is not in the correct position in the breast | No |
|  |  | Yes |
| Capsule (T85.4) | Perioperative status at reoperation: Capsule (T85.4). Dense connective tissue capsule formed around the implant and requiring surgical intervention (Baker III, IV) | No |
|  |  | Yes |
| Seroma/Exudate (T81.8) | Perioperative status at reoperation: Accumulation of wound fluid in the prosthetic cavity | No |
|  |  | Yes |
| Hematoma | Perioperative status at reoperation: Accumulation of blood in, or outside, the prosthetic cavity | No |
|  |  | Yes |
| Permanent extract of the implant | Surgical intervention at reoperation: Implant is extracted without insertion of a new implant | No |
|  |  | Yes |
| Reinsertion of current implant | Surgical intervention at reoperation: Implant is removed, and after treatment, the same implant is reinserted | No |
|  |  | Yes |
| New insertion of implant following previous implant extraction | Surgical intervention at reoperation: A new implant is inserted following previous implant extraction, for instance after an infection or other conditions where breast tissue needs to heal for several months without implant | No |
|  |  | Yes |
| Implant change | Surgical intervention at reoperation: A new implant is inserted during the same session as current implant is extracted | No |
|  |  | Yes |
| Capsule cleavage | Surgical intervention at reoperation: Incision of the capsule (capsulotomy) in one or multiple quadrants | No |
|  |  | Yes |
| *En bloc* resection | Surgical intervention at reoperation: The implant and capsule are removed as a unit without incision of capsular tissue, regardless of the indication and diagnosis | No |
|  |  | Yes |
| Total capsule removal | Surgical intervention at reoperation: The entire capsule (total capsulectomy) is removed including the capsule in contact with the thoracic wall | No |
|  |  | Yes |
| Partial capsule removal | Surgical intervention at reoperation: Parts of the capsule are removed (partial capsulectomy) | No |
|  |  | Yes |
| Insertion of a net or an acellular dermal matrix | Surgical intervention at reoperation: Insertion of net or acellular dermal matrix in the current operation | No |
|  |  | Yes |

**Supplementary Table 2. Cumulative incidence of capsular contracture requiring surgery through one to five years.** The cumulative incidence of capsular contracture requiring surgery was analysed using Kaplan-Meier estimates through one to five years, respectively.
Abbreviations: CI=confidence interval.

| **Supplementary Table 2. Cumulative incidence of severe capsular contracture requiring surgery through one to five years** | | |
| --- | --- | --- |
| Interval (years) | Number of capsular contractures/number at risk | Cumulative incidence (95% CI), % |
| 0–1 | 19/1 095 | 1.88 (1.21–2.94) |
| 1–2 | 13/902 | 3.43 (2.43–4.82) |
| 2–3 | 3/741 | 3.86 (2.78–5.35) |
| 3–4 | 1/593 | 4.04 (2.92–5.58) |
| 4–5 | 3/482 | 4.74 (3.43–6.52) |
